# The Smoke Nuisance

**Published:** 1905-12-09

**Authors:** 


### The Hospital.

A Journal of

The Medical Sciences and Hospital Administration.

Vol. XXXIX.—No. 1,002. SATURDAY, DECEMBER 9, 1905.

### THE SMOKE NUISANCE.

THE Conference on Smoke Abatement, to be held under the presidency of Sir Oliver Lodge, on the 12th, 13th, 14th, and 15th instant at the Hall of the Royal Horticultural Society, Westminster, will, we hope, bring together many earnest supporters of the reform movement which may be said to owe its origin to Sir William Richmond, R.A., and which deserves the active assistance not only of artists, and of all those who are interested in beauty, but also, and in a still greater degree, of those who desire the preservation of health. We cannot be quite sure, indeed, whether on the score of beauty there may not be another side to the question; and whether we may not be indebted to the smoke of the metropolis for some of the most artistic of its aspects. The "Pool," as the late Mr. Vicat Cole painted it, and the colours of suburban sunsets, are largely dependent upon the varied reflection and absorption of light effected by smoke particles; and hence, when we find a Royal Academician placing himself in the van of a much needed reform, we may feel sure that the case in favour of it must indeed be a strong one. It is so strong that we greatly doubt whether it would be possible to find any additional aspect of the case in which the smoke of our great towns is other than an unmixed evil, and we are glad to observe that the Conference will be attended by numerous delegates from metropolitan and provincial authorities and societies. Sir John Primrose, Sir William Preece, Sir William Richmond, Sir George Livesey, and Sir Shirley Murphy have accepted office as Vice-Presidents; and the Duke of Fife will take the chair at the inaugural meeting at 8.30 on Tuesday evening, when the President will deliver an address on the general subject of combustion reform. On Wednesday morning the Conference will assemble for business, to hear and discuss papers on domestic smoke abatement; and, after luncheon, will visit the Chelsea generating station of the underground electric railways. On Thursday the subject of the day will be factory and trade smoke abatement, and a visit will be paid to the gasworks of the South Metropolitan Company. On Friday the subject of the day will be administration, legislation, and necessary reforms, and the visit will be to the Abbey Mills pumping station of the London sewage works. Among the papers promised is one by Mr. Rigg, on behalf of the Royal Botanic Society of London, on "The destructive effect of smoke in relation to plant life," and another on the same subject by Miss M. Agar, representing the Metropolitan Gardens Association. Dr. Rideal will deal with "The Acids of Smoke," and the Hon. Rollo Russell, Fellow of the Royal Meteorological Society, with "The artificial production of persistent fog."

It is a matter of common knowledge that the possibilities of horticulture in the metropolis are now limited, and the suburban garden is becoming a thing of the past. The fortunate or diligent person who succeeds in growing a few flowers is unable to pluck them without soiling his fingers; and the carbonaceous deposit which is visible on the upper surfaces of the leaves soon reaches also the under surfaces, and chokes the stomata upon which the plant is dependent for its respiration. If this be so with plants, it must to some extent be the same with regard to human beings; and we regret not to find in the programme of the Conference any reference to the effects of smoke pollution of the atmosphere upon the health of mankind. It would be within the power of the Registrar-General to furnish some very striking evidence of the effects of a few days of smoke-laden fog upon human mortality; effects which, whenever a period of such fogs has occurred, are always abundantly manifest in the obituaries of the newspapers, although these deal only with comparatively sheltered classes of the community. It is impossible to doubt that an inquiry including the whole of the population would show a black fog to be almost as serious a matter as an epidemic; and, precisely as with an epidemic, many who do not immediately swell the ranks of mortality must be left more or less crippled by the injury which they have sustained. We hope that the success of the coming Conference will be such as to require that it should be periodically repeated, and that, in any future gatherings of the same kind, the influences of smoke upon human health will be more fully dealt with than seems to be intended upon the present occasion. There must be many physicians, not only in the metropolis, but also in the great manufacturing towns, who would have an abundance of material for instructive papers, and who could illustrate the consequences of inhaling sulphur and carbon dust by examples derived from large experience. In the meanwhile there is only too much reason for denouncing the nuisance on the grounds of the pecuniary loss which it inflicts upon the community, and especially upon the trading community, which is usually more sensitive to arguments of such a kind than to any which are based only upon the health of other people. The indirect losses due to impeded traffic, the direct injury done by unnecessary smoke to clothing, to decorations, to silver, to stocks of goods of every description, cannot be imagined to amount, in this country, to less than a sum countable by millions, and it must not be forgotten that it all arises from sheer waste, from the dissipation of unconsumed fuel which should be made to yield its quota of force to the consumer, but which is now left to be useless by reason of its escape from combustion.

